# Anharmonic effects on the dynamical stability of Ce–Co–Cu intermetallic ternary compounds

**DOI:** 10.1039/d5ra09680d

**Published:** 2026-03-17

**Authors:** Wei-Shen Tee, Weiyi Xia, Rebecca Flint, Cai-Zhuang Wang

**Affiliations:** a Ames National Laboratory, U.S. Department of Energy, Iowa State University Ames Iowa 50011 USA; b Department of Physics and Astronomy, Iowa State University Ames Iowa 50011 USA

## Abstract

Ce-based intermetallic compounds are of growing interest for their potential applications in energy-efficient permanent magnets. While recent machine learning and DFT studies predicted several new Ce–Co–Cu ternary compounds to be stable at *T* = 0 K, their dynamical stability requires further investigation. We show that first-principles harmonic phonon calculations predict imaginary vibrational modes for some structures, suggesting they are dynamically unstable at 0 K. However, *ab initio* molecular dynamics (AIMD) simulations reveal that these structures are stable at finite temperatures. Vibrational density-of-states and phonon modes calculated at finite temperature through the AIMD simulations suggest that anharmonic interactions are important in stabilizing these predicted Ce–Co–Cu intermetallic compounds.

## Abstract 2

A predicted Ce–Co–Cu compound shows high-temperature dynamical stability yet exhibits a double-well potential energy landscape below 200 K.

## Graphical abstract

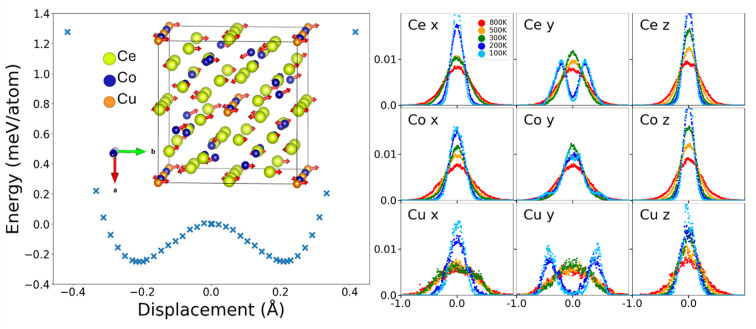

## Introduction

The Ce–Co–Cu ternary system is of strong interest for exploration due to its potential to address both practical and fundamental challenges in materials design. First, cerium (Ce) is a more abundant and cheaper alternative to critical rare-earth elements like neodymium (Nd),^1^ samarium (Sm), and dysprosium (Dy), which are commonly used in high-performance permanent magnets. Replacing these critical elements with Ce could improve the sustainability and economic viability of magnet technologies. Second, Ce exhibits rich and tunable electronic and magnetic behaviors due to its ability to fluctuate between Ce^3+^ and Ce^4+^ valence states,^2^ making it an ideal platform for studying strongly correlated phenomena. Finally, despite the known binary phases in the Ce–Co and Ce–Cu systems,^3^ there is a notable absence of experimentally confirmed ordered ternary Ce–Co–Cu compounds. This lack of structural knowledge presents an opportunity for computational discovery and insight into the stability mechanisms that may govern their formation.

Discovery of new materials with emergent physical properties, such as superconductivity and exotic topological or magnetic states, has been a subject of intensive research for decades.^4^ Traditionally, most new materials were discovered through trial-and-error experiments, which require substantial time and effort. Rapid advances in modern computational hardware/software, machine learning, and advanced synthesis methods offer exciting opportunities for accelerating the pace of materials discovery. In the past few decades, several efficient new material searching methods have been developed, including random structure search,^5^ CALYPSO,^6^ genetic algorithm,^7,8^ adaptive genetic algorithm (AGA),^9–15^ machine-learning guided framework,^15–19^*etc.* These computational approaches have contributed significantly to the advances in new materials discovery. However, it should be noted that these computational methods only predicted the thermodynamic stability of the phases/structures at *T* = 0 K. Further validations of the dynamical stability for the predicted structures are often necessary.

Currently, most computational verification of dynamical stability of the predicted structures is based on the calculations of phonon dispersion relations using harmonic (or quasi-harmonic) approximations.^14,18^ The appearance of imaginary phonon modes in such calculations is often used as a criterion to classify the structure as dynamically unstable.^20^ However, many materials exhibit imaginary phonon modes at *T* = 0 K in harmonic calculations but are stable at finite temperatures. For example, group IV elements' body-centered cubic (BCC) structure, *e.g.*, Ti, Zr, and Hf, have imaginary frequencies at 0 K but can exist at high-temperature conditions.^21^ Similarly, perovskites such as KTaO_3_ ^22^ and CsPbBr_3_ ^23^ exhibit soft modes in harmonic calculations that are dynamically stabilized through strong anharmonic lattice fluctuations.

Numerous studies indicate a wide array of materials exhibiting anharmonicity. These include: (1) Perovskites and related frameworks, such as oxide and halide perovskites including KTaO_3_,^22^ CaSiO_3_,^24–26^ MgSiO_3_,^27^ KNbO_3_, NaNbO_3_,^28^ SrTiO_3_,^29^ CsPbBr_3_,^23,30^ CH_3_NH_3_PbI_3_,^31^ and other cubic perovskites surveyed in systematic DFT studies.^32,33^ These compounds exhibit soft phonon modes, quantum fluctuations, and temperature-driven instabilities. (2) Thermoelectric and chalcogenide systems like PbTe, SnTe, and SnSe,^34–38^ where strong anharmonic phonon interactions suppress thermal conductivity and alter phonon dispersion. (3) Superconductors and quantum materials, including Y_2_C_3_,^39^ Nb_2_S,^40^ H_3_S,^41^ PdH,^42^ LuH_3_,^43^ and LaH_10_,^44^ in which anharmonic effects play a crucial role in mediating superconductivity. (4) High-pressure or high-temperature phases, such as Ti, Zr, and Hf,^21,45,46^ simple-cubic Ca,^47^ Be,^48^ and Fe,^49,50^ where lattice instabilities or quartic phonon potentials emerge under temperature or pressure changes. (5) Surfaces and low-dimensional materials, like Cu (110)^51^ and Be (0001),^52^ where surface phonon broadening is strongly temperature-dependent due to anharmonicity. These categories are not exhaustive but highlight the diversity of materials classes where anharmonic effects are known to play a critical role.

Several important factors beyond the harmonic interactions affect the dynamical stability of the crystalline structures.^20^ Firstly, atoms do not sit in harmonic potential wells but in anharmonic potential wells in real materials. Because of the anharmonic interactions, the higher order expansions in the total energy cost with respect to the atomic displacement cannot be ignored, thus affecting the calculated phonon frequencies even at *T* = 0 K. Secondly, atoms do not sit in a potential created by static neighbors but instead by dynamic neighbors at finite temperatures. As temperature increases, atoms explore a larger part of the energy landscape through a larger vibrational amplitude, which means the atoms can access larger numbers of microstates and thus larger the system's entropy. A larger entropy with a higher temperature can significantly decrease the Gibbs free energy (*G* = *U* − TS + PV). Therefore, to assess the dynamical stability of a structure, one also needs to consider the effects of anharmonic interactions and the contribution of entropy at finite temperatures.

In this paper, we further demonstrate this point of view by studying the dynamical stability of the newly predicted Ce–Co–Cu ternary compounds^15^ through *ab initio* molecular dynamics (AIMD) simulations.^27,28,53–57^ Whereas harmonic phonon frequencies are often used as a stability filter, at the risk of discarding intrinsically anharmonic new materials, AIMD provides a more versatile approach for demonstrating the dynamic and thermal stability of newly discovered materials. By allowing large atomic displacements at finite-temperature, AIMD automatically samples the anharmonic effects and can be performed over a broad temperature range, yielding insights unavailable to harmonic approximations. AIMD is also computationally efficient for low symmetry systems: it avoids the many independent displacements required to build the full force-constant matrix in finite-displacement phonon calculations. From the resulting trajectories, we can obtain atomic probability densities; pair-correlation functions that reveal atomic neighbor relationships; and velocity autocorrelation functions (VACF), whose Fourier transforms give the vibrational density of states (DOS). Because the VACF formalism^24,25,48,49,57–59^ does not assume the underlying Hamiltonian, it captures anharmonic interactions to all orders, providing a powerful avenue to probe higher-order lattice effects beyond the reach of harmonic theory.

## Methods and simulations details

The first-principles density functional theory (DFT) calculations were performed using Vienna *ab initio* simulation package (VASP)^60–62^ with projector-augmented wave (PAW) pseudopotentials^63^ and Perdew–Burke–Ernzerhof (PBE) exchange–correlation energy functional.^64^ Converged results are obtained with a kinetic energy cutoff for the plane wave basis of 520 eV and a *k*-point grid with a spacing of 0.2 Å^−1^. The equilibrium structure is optimized until all forces are below 0.01 eV Å^−1^. DFT-PBE provides reasonable, low-cost *ab initio* description of structural and dynamical stability for many Cerium compounds. Since the main purpose of our present study is to compare dynamical stability from harmonic calculations *versus* anharmonic finite-temperature simulations, using the same DFT-PBE method for both calculations would be appropriate.

The harmonic phonon calculations are performed using the finite displacement method implemented in the Phonopy code,^65,66^ based on spin-polarized forces obtained from VASP. The supercells, consisting of 128 atoms for CeCoCu_2_, 200 atoms for Ce_10_Co_11_Cu_4_, and 80 atoms for Ce_12_Co_7_Cu, are employed to capture the interatomic interactions. Due to computational constraints, these sizes may not be sufficient to achieve fully converged phonon spectra.

The *ab initio* molecular dynamics (AIMD) simulations were carried out using VASP without spin polarization since the compounds studied have been reported to be nonmagnetic at 0 K.^15^ The same supercells used in the harmonic phonon calculations are used for CeCoCu_2_, Ce_10_Co_11_Cu_4_, and Ce_12_Co_7_Cu. A lower kinetic energy cutoff of 300 eV was used to expedite the simulations. A time step of 3 femtoseconds (fs) was employed in the AIMD simulations. To validate this choice, convergence tests were performed using time steps of 1 fs, 2 fs, and 3 fs for Ce_10_Co_11_Cu_4_ (see Fig. S3 in the SI). The resulting atomic trajectories exhibited no significant qualitative or quantitative differences across key indicators of dynamical stability, including total energy fluctuations, temperature control, atomic displacements, and the vibrational density of states (VDOS). Note that the system contains heavy elements (Ce, Co, and Cu); therefore, there are no high-frequency vibrational modes typically associated with light atoms such as hydrogen, allowing for the use of a larger time step (3 fs) in the AIMD simulations without compromising accuracy.

The supercells used in the AIMD simulations are the same as those used in the harmonic phonon calculations. For the Ce_12_Co_7_Cu, the 80-atom supercell with lattice dimensions of approximately 11.5 × 11.5 × 12.8 Å^3^. Although this supercell contains a relatively modest number of atoms, its linear dimensions exceed those commonly employed in *ab initio* molecular dynamics simulations (typically around 10 × 10 × 10 Å^3^ with periodic boundary conditions). Finite-size effects on vibrational properties may exist, particularly on long wavelength dynamical behavior, but the effects on the short wavelength dynamical behavior should be minimal. Similar finite size effects are also in the harmonic phonon calculations. It is reasonable to compare the phonons from harmonic calculations and anharmonic AIMD simulations using the same supercell size and periodic boundary conditions to elucidate the anharmonic effects on the stability of the structures.

For each temperature, the system was first equilibrated for 3 ps in an NVT (constant number of particles, volume, and temperature) ensemble with temperature controlled by the Langevin thermostat. Then, the simulation is switched to an NVE (constant number of particles, volume, and energy) ensemble for more than 10 ps to collect the trajectories of the atoms for structural and dynamical analysis, including the analysis of atomic distribution functions, pair correlation functions, and velocity autocorrelation functions. The probability distribution of atomic displacement relative to the perfect structure at various temperatures provides insight into the preferred atomic positions at finite temperatures. These preferred positions are directly influenced by the shape of the potential well, as atoms tend to occupy lower-energy positions. To mimic conditions closer to experimental reality, we have also checked the results using an NPT (constant number of particles, pressure, and temperature) ensemble with temperature controlled by the Langevin thermostat.

The pair correlation function *g*_AB_ is defined as a measure of the probability of finding particles B at *r* away from A, relative to that for an ideal gas:1
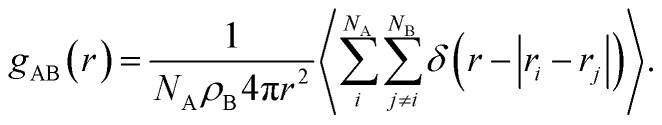
Here, *N*_A_ is the number of particles A, *ρ*_B_ is the average density of particles B, 
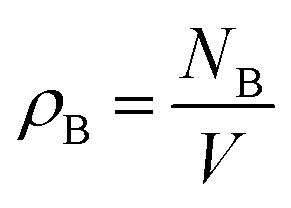
. In general, *g*_AB_ = 1 describes data with no structure (ideal gas). We then compare 3 pair correlation functions (PCF): PCF of the initial structure, PCF of the averaged structure, and time-averaged PCF. PCF of the initial structure represents the structural properties of the original structure at the initial time step. The purpose of this PCF is to provide a baseline reference, reflecting the structural order and symmetry at the beginning of the simulation. PCF of the averaged structure is calculated using the averaged atomic positions obtained from all structures at each time step. If the atom is vibrating around an equilibrium position, the positive displacement will cancel the negative displacement, and the averaged structure should be identical to the initial structure. The PCF of the average structure serves as an indicator of structural stability and anharmonic effects; if it closely resembles the initial structure, the material retains its original symmetry and order, whereas deviations suggest significant atomic rearrangements or anharmonic effects. Time-averaged PCF is calculated by averaging the PCFs of all configurations across the entire simulation trajectory. It reflects the time-averaged pair correlation over the whole simulation. Time-averaged PCF captures dynamic changes of the structure, including both equilibrium and transient states.

Velocity trajectories can be extracted from the AIMD simulation at each temperature and can generally be transformed into the phonon density of state (DOS) and phonon dispersion relation. The phonon DOS at each temperature can be calculated by taking the Fourier transform of the velocity autocorrelation function (VACF),^24,25,48,49,57–59^ defined as,2
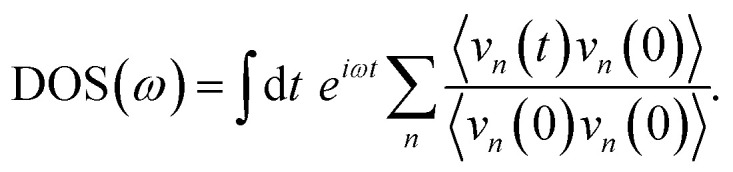
where 〈〉 is an ensemble average, and *ν*_*n*_(*t*) is the velocity trajectory of atom *n* at time step *t*. Further projection of the velocity autocorrelation function onto each *k* point in the Brillouin zone, we can get the phonon spectral intensity at that temperature:3
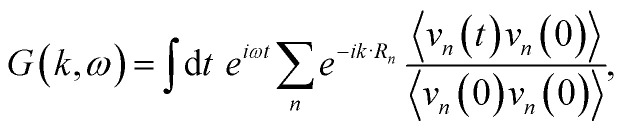
where *R*_*n*_ is the lattice position of atom *n*.

## Results and discussions

In our previous works,^15^ we predicted five stable compounds (Ce_3_Co_3_Cu, CeCoCu_2_, Ce_12_Co_7_Cu, Ce_11_Co_9_Cu, Ce_10_Co_11_Cu_4_) with formation energies below the convex hull, and two Co-rich low-energy compounds (Ce_4_Co_33_Cu, Ce_4_Co_31_Cu_3_) with formation energies slightly above the convex hull. No imaginary vibrational frequencies were observed in the phonon dispersions of Ce_3_Co_3_Cu, CeCoCu_2_, Ce_11_Co_9_Cu, Ce_4_Co_33_Cu, and Ce_4_Co_31_Cu_3_, indicating the dynamic stability of these structures at 0 K. For Ce_10_Co_11_Cu_4_ and Ce_12_Co_7_Cu, we obtain imaginary phonons from the harmonic calculations, which suggests that the structures might be dynamically unstable. However, harmonic calculations alone may not fully reflect the actual stability of the structure, as anharmonic interactions can play an important role in dynamical stability, especially at finite temperatures.

To see the effects of anharmonic interactions and temperature on the vibrational properties and dynamical stability, we select three representative structures, *i.e.*, CeCoCu_2_, Ce_10_Co_11_Cu_4_, and Ce_12_Co_7_Cu, from our previous work for more detailed comparative studies. Under the harmonic approximation, CeCoCu_2_ is dynamically stable without any imaginary phonon modes, Ce_10_Co_11_Cu_4_ exhibits some imaginary modes around high symmetry *k*-points (*Z*, *D*, *E* and *C*_2_), while Ce_12_Co_7_Cu has many imaginary modes around several *k*-points.

### CeCoCu_2_ phase

**Fig. 1:**
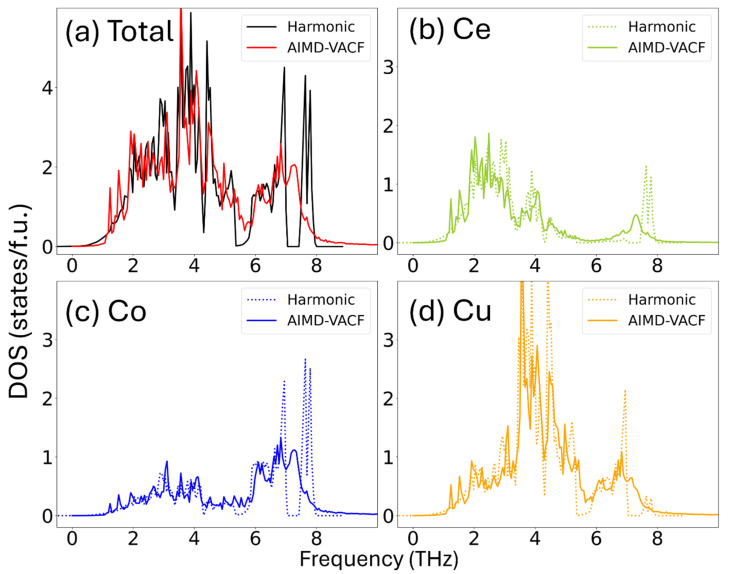
Total and elemental-projected vibrational density of states (DOS) of CeCoCu_2_ calculated using the harmonic approximation at 0 K are compared with those from AIMD simulations at 500 K using the velocity autocorrelation function (VACF) method. (a) Total DOS. (b–d) Partial DOS projected onto Ce, Co, and Cu atoms, respectively.

The total and projected phonon density of states (DOS) of the stable CeCoCu_2_ phase obtained from harmonic calculation at *T* = 0 K^15^ and AIMD simulations at *T* = 500 K are shown in Fig. 1(a)–(d). The two DOS profiles are in good agreement: both exhibit phonon states extending up to 8 THz and share a prominent peak at 4 THz corresponding to the Cu atoms. The close similarity between the results demonstrates the consistency between the harmonic and AIMD-VACF approaches for stable materials. The discrepancies between the two DOS profiles (*e.g.*, the sharp peaks in the harmonic DOS at *T* = 0 K are washed out in the AIMD simulations at 500 K) can be attributed to the inclusion of anharmonic effects in the VACF-based calculation, which are not accounted for within the harmonic approximation framework.

### Ce_10_Co_11_Cu_4_ phase

**Fig. 2:**
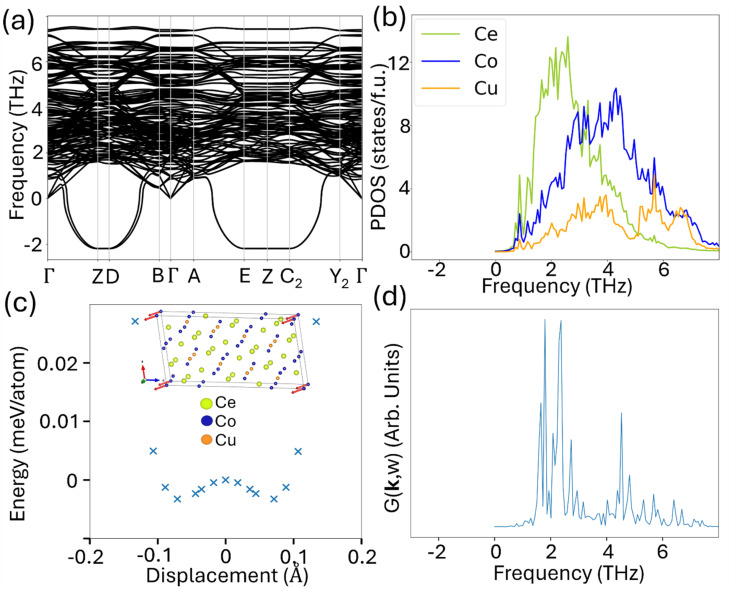
(a) Phonon dispersion relation of Ce_10_Co_11_Cu_4_. (b) Projected density of states (PDOS) at 500 K (NVT-NVE) obtained from AIMD. (c) The energy of the imaginary phonon mode at the *Z* point is shown along with its corresponding eigenvector, and the displacement magnitude reflects the largest atomic contribution within the eigenvector. (d) The corresponding phonon spectral intensity of the Z mode from the AIMD simulation at 500 K.

Imaginary phonon frequencies are observed around the *Z*, *D*, *E*, and *C*_2_ points for this compound from the harmonic calculation as shown in Fig. 2(a). To examine whether the imaginary vibrational modes originate from the force convergence criterion of 0.01 eV Å^−1^, we re-relax the structure using tighter force tolerance (≤0.001 eV Å^−1^). The refined structure is then used for the phonon calculation. The imaginary phonon modes around the *Z*, *D*, *E*, and *C*_2_ high-symmetry points persist even with the tighter force convergence criterion. However, in the projected phonon density of states (PDOS), computed using the velocity autocorrelation function (VACF) approach from AIMD simulations at 500 K, as shown in Fig. 2(b), no signs of diffusive behavior are observed. The PDOS obtained from the AIMD simulation suggests that this structure is dynamically stable at 500 K, presumably due to the anharmonic effects. The PDOS obtained from AIMD closely resembles that from the harmonic approximation, with the key difference being the absence of the imaginary phonon mode in the AIMD results. When atoms are displaced along the eigenvectors corresponding to the imaginary vibrational mode at the *Z* point, a shallow double-well potential is observed, with a well depth of approximately 0.003 meV per atom and minima located around 0.07 Å, as illustrated in Fig. 2(c), indicating the presence of weak anharmonicity. In this plot, the energy *versus* displacement is calculated by performing static total energy calculations for a series of structures generated by displacing atoms in both positive and negative directions along the mode *Z* eigenvector to evaluate the system's energy response to the distortion. As the anharmonic interactions are included through the AIMD, no soft modes are observed at the *Z* point at 500 K, as can be seen from the vibration spectra at the *Z* point from AIMD simulations shown in Fig. 2(d).

**Fig. 3:**
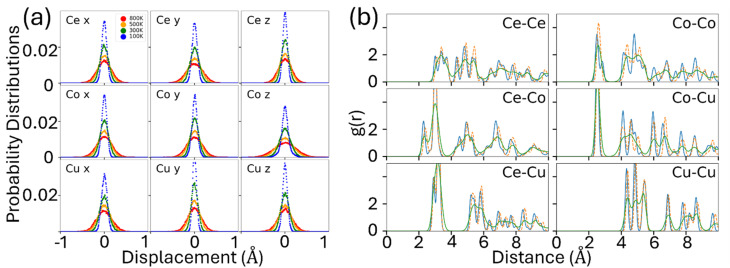
(a) Probability distribution of atomic displacements for the ideal Ce_10_Co_11_Cu_4_ at various temperatures (NVT-NVE). (b) Pair correlation function (PCF) of each element pair at 500 K (NPT). The blue line is the PCF of the initial structure, the orange line is the PCF of the averaged structure, and the green line is the time-averaged PCF of all time steps.

The dynamical stability of this structure is also confirmed by the statistics of the atomic position in the AIMD simulations, as shown in Fig. 3. As shown in the probability distribution in Fig. 3(a), atomic vibrations are centered around equilibrium positions. At lower temperatures, atomic displacements remain minimal, while increasing temperature leads to larger vibrational amplitudes due to enhanced thermal energy. To further validate structural stability, we performed an NPT simulation at 500 K. The pair correlation function (PCF) of the averaged structure (orange line) and the time-averaged PCF (green line), shown in Fig. 3(b), closely match the initial structure's PCF (blue line). These findings collectively confirm that the structure remains stable up to 800 K despite the presence of imaginary phonon modes at 0 K.

### Ce_12_Co_7_Cu phase

**Fig. 4:**
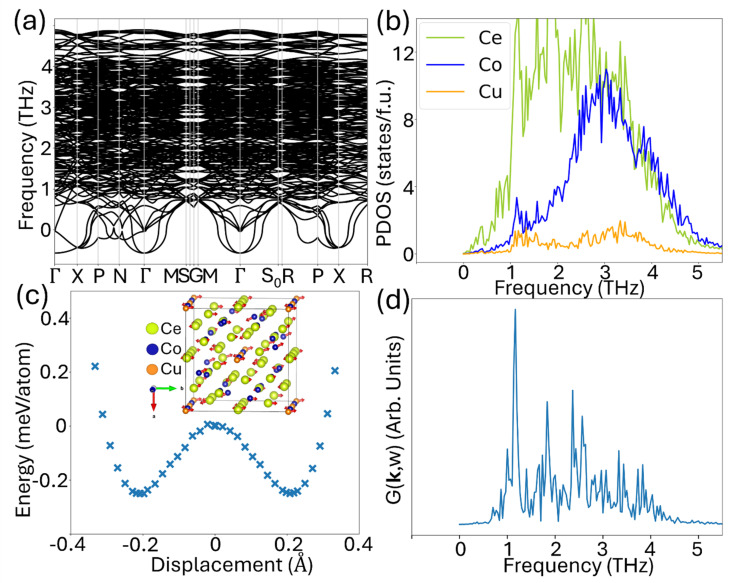
(a) Phonon dispersion relation of Ce_12_Co_7_Cu. (b) Projected density of states (PDOS) at 500 K (NVT-NVE) obtained from AIMD. (c) The energy of the imaginary phonon mode at the *Γ* point is shown along with its corresponding eigenvector, and the displacement magnitude reflects the largest atomic contribution within the eigenvector. (d) The corresponding phonon spectral intensity of the X mode from the AIMD simulation at 500 K.

Fig. 4(a) displays the phonon dispersion relation of Ce_12_Co_7_Cu from *T* = 0 K harmonic calculation, where imaginary phonon modes indicate the structure is dynamically unstable under the harmonic approximation. Again, the PDOS obtained from AIMD simulation at 500 K, as shown in Fig. 4(b), suggests that this structure is dynamically stable at 500 K. We note that strong imaginary modes would lead to a lower-energy structure if atoms are displaced along the corresponding eigenvector of the soft modes. To explore this, we displaced the atoms along the eigenvector at the *Γ* point, as illustrated in the insert of Fig. 4(c). The associated energy profile with displacement amplitude obtained from our DFT calculation is also plotted in Fig. 4(c). The resulting energy landscape reveals a double-well potential along the *y*-direction, with the minima located approximately 0.2 Å from the original atomic positions and a well depth of about 0.25 meV per atom. While the double well potential is responsible for the soft modes at *T* = 0 K, the projected density of states (PDOS) in Fig. 4(b) and the phonon spectral intensity of the X mode in Fig. 4(d) from the AIMD simulation at 500 K exhibit no evidence of diffusive behavior, indicating this structure is stable at finite temperatures. Notably, the spectral intensity of the X mode remains positive across all frequencies, indicating that this phonon mode—initially imaginary at 0 K—can be stabilized by anharmonic interactions. Furthermore, the AIMD and harmonic PDOS match well, except that the AIMD result lacks the imaginary mode.

**Fig. 5:**
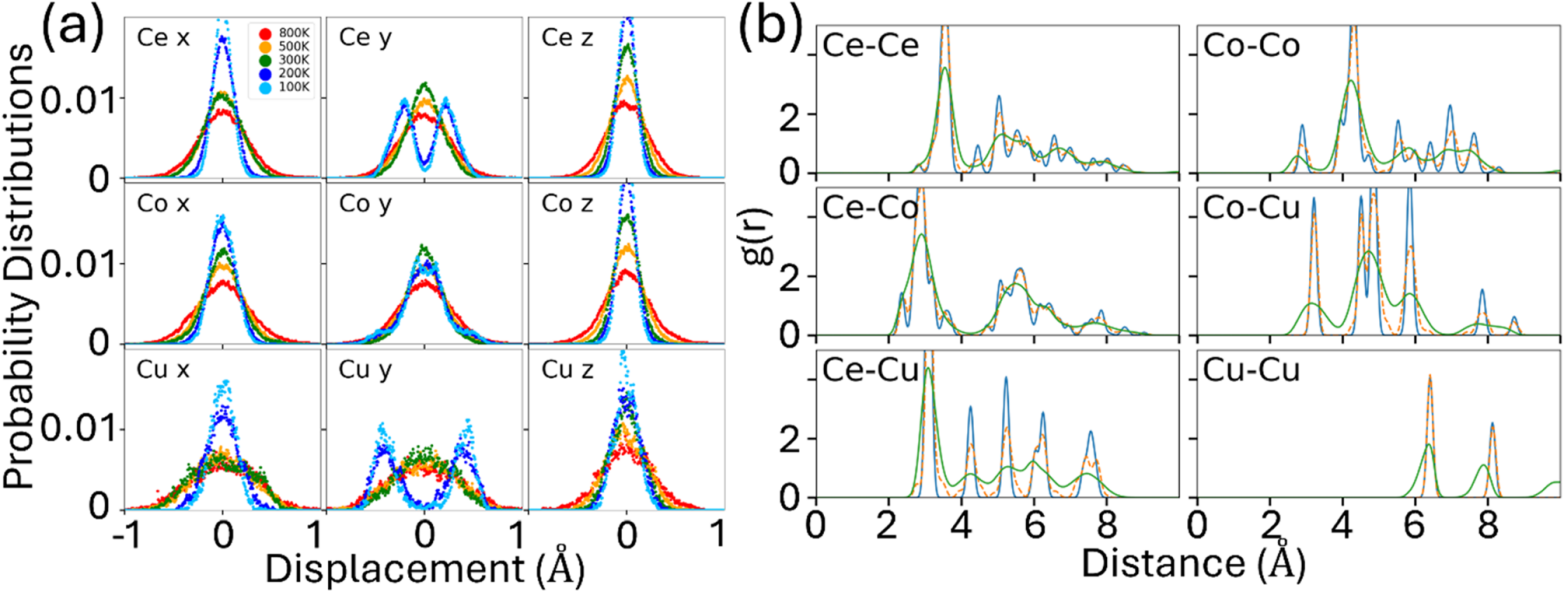
(a) Probability distribution of atomic displacements for the ideal Ce_12_Co_7_Cu at various temperatures (NVT-NVE). (b) Pair correlation function (PCF) of each element pair at 500 K (NPT). The blue line is the PCF of the initial structure, the orange line is the PCF of the averaged structure, and the green line is the time-averaged PCF of all time steps.

The dynamical stability of this structure at finite temperature can also be seen from the statistical analysis of temperature-dependent atomistic trajectories from the AIMD simulations. Fig. 5(a) shows the probability distribution of atomic displacements relative to the equilibrium positions at various temperatures. At 300 K, 500 K, and 800 K, atoms are distributed symmetrically around a peak at the initial equilibrium positions, respectively. At 100 K, vibrations along the *x*- and *z*-directions also peak at the initial equilibrium position. However, along the *y*-direction, atoms Ce, Co, and Cu exhibit bimodal distribution, with split peaks located at 0.22 Å, 0.07 Å, and 0.45 Å, respectively, away from the initial equilibrium position, indicating occupation of two distinct positions within a double-well potential. At low temperatures, the thermal energy is insufficient to overcome the barrier between the wells, so atoms are trapped in either the left or right potential minimum.

By examining the time-dependent atomic trajectories from the AIMD simulation at 100 K and 200 K, we observed that the Ce and Cu atoms are relaxed away from the high symmetry saddle point and randomly reside in one potential well or another with almost equal probability. Atomic hopping between the two wells is rare (quantum tunneling is not considered in AIMD simulations). Therefore, the structure below 200 K can be characterized as thermally average mixtures of locally distorted configurations. As the temperature increases, hopping events become increasingly frequent due to the very small energy barrier (about 0.25 meV per atom) between the two wells. At 300 K and above, the atomic distributions show a single peak at the high symmetry lattice points, the system thus can be considered as a dynamically stabilized single phase. Therefore, crossover from thermally average mixtures of locally distorted configurations to a dynamically stabilized single phase at a temperature between 200 and 300 K is observed for this complex compound.

This bimodal behavior at low temperature aligns with predictions from harmonic analysis, wherein modes involving *y*-direction displacements appear as imaginary. As the temperature rises, atoms gain sufficient kinetic energy to overcome the wells, effectively averaging the potential landscape into a single-well shape. Additional NPT simulations confirm these findings, yielding pair correlation functions (PCFs) consistent with those obtained under the NVT-NVE ensemble, as shown in Fig. 5(b). These results demonstrate that the structure remains dynamically stable, with the observed imaginary phonons at 0 K arising from the limitations of the harmonic approximation in capturing double-well potentials. Similar double-well potential and temperature-driven stabilization mechanisms have been reported in perovskites and thermoelectric,^22,23,25,28,32,35,50,67,68^ providing context for the observed behavior in Ce-based intermetallics.

## Conclusions

In conclusion, we have studied the dynamical stability of three proposed Ce-based ternary compounds (CeCoCu_2_, Ce_10_Co_11_Cu_4_, and Ce_12_Co_7_Cu) using harmonic phonon calculations at 0 K and *ab initio* molecular dynamics (AIMD) simulations at various temperatures.

While the CeCoCu_2_ phase is stable under both methods, the Ce_10_Co_11_Cu_4_ and Ce_12_Co_7_Cu phases show imaginary modes in harmonic approximation, suggesting instability. For Ce_10_Co_11_Cu_4_, the potential energy landscape exhibits a shallow double-well potential with a depth of only 0.003 meV per atom and minima located at ±0.07 Å, suggesting very weak anharmonicity, close to harmonic stability. For Ce_12_Co_7_Cu, a more pronounced double-well is observed with a depth of 0.25 meV per atom and minima at ±0.2 Å, indicating stronger anharmonic effects and a significant breakdown of the harmonic approximation. These observations are further supported by AIMD simulations: for Ce_10_Co_11_Cu_4_, the atomic displacement distributions remain unimodal even at 100 K, and for Ce_12_Co_7_Cu, clear bimodal distributions emerge at both 100 K and 200 K. AIMD results confirm that all three phases are dynamically stable up to 800 K due to anharmonic effects. In both Ce_10_Co_11_Cu_4_ and Ce_12_Co_7_Cu phase, double-well potential leads to 0 K instabilities that are mitigated at higher temperatures. Thermal fluctuations allow atoms to transition between the wells, effectively averaging the potential landscape and restoring dynamical stability.

While our discussion of anharmonic effects is qualitative, we have supplemented it with quantitative characterizations of atomic behavior that directly reflect the impact of anharmonicity. Specifically, we present atomic displacement distributions extracted from AIMD simulations, which offer a statistical description of atomic motion at finite temperatures. These distributions provide direct insight into the atomic fluctuations associated with anharmonic potential energy landscapes. In addition, we analyze real-space atomic trajectories, vibrational densities of states (VDOS), and pair correlation functions to further support the characterization of dynamical behavior. Together, these data provide a physically grounded picture of how anharmonicity contributes to the finite-temperature stability of the predicted materials.

The finite-temperature dynamical behavior of the studied compounds is evaluated using several complementary indicators derived from the AIMD simulations. First, the atomic trajectories do not exhibit long-range diffusion, suggesting that the systems remain structurally bounded over 10 ps. Second, both the instantaneous temperature and total energy fluctuate stably around their target values over simulations exceeding 10 ps, indicating numerically stable finite-temperature sampling.^15^ Third, the short-range structural correlations are maintained throughout the simulations, as reflected by well-defined pair correlation functions, implying the preservation of local atomic order. Finally, the vibrational spectra obtained from velocity autocorrelation functions exhibit positive spectral intensity and their close similarity to the harmonic results demonstrates the overall consistency between the harmonic and AIMD-VACF approaches. Collectively, these observations support the conclusion that the investigated structures exhibit finite-temperature dynamical stability within the timescales and conditions considered here.

These results demonstrate that imaginary phonon modes at 0 K do not necessarily indicate instability at finite temperatures. AIMD provides a more comprehensive framework for evaluating thermal stability, as it inherently captures both anharmonic effects and thermal fluctuations. Overall, our findings emphasize the importance of going beyond harmonic phonon calculations when searching for new materials to avoid overlooking promising materials that exhibit anharmonic stability at finite temperatures.

As a complementary approach, the temperature-dependent effective potential (TDEP) method^23,37,69^ can be used to study anharmonic systems by fitting renormalized force constants from finite-temperature AIMD trajectories. It should be noted that AIMD becomes less reliable as the temperature approaches 0 K,^47^ as its classical Newtonian mechanics framework fails to capture the quantum effects that dominate in the low-temperature limit. In these cases, the stochastic self-consistent harmonic approximation (SSCHA) is a more suitable method,^41,70^ as it incorporates quantum statistics and can accurately capture anharmonic behavior beyond the harmonic approximation, even as the temperature approaches zero.

While anharmonic effects and dynamical stability are essential for understanding the physical behavior of materials, they alone do not guarantee experimental synthesizability.^71^ The synthesis of materials is governed by both thermodynamics and kinetic factors. Thermodynamic factors – such as having a formation energy on or below the convex hull (*e.g.*, Δ*E*_hull_ = 0), the absence of imaginary phonon modes, and dynamic stability confirmed by molecular dynamics simulations – are commonly used to determine whether a material is energetically and dynamically stable. However, even if these thermodynamic conditions are satisfied, synthesis may still be challenging due to unknown formation pathways.^72^ Kinetic factors – such as reaction pathways,^73^ activation barriers,^74^ and experimental constraints – play a crucial role in synthesizing materials. In many cases, multiple phases may compete during synthesis, and without detailed kinetic information, it can be challenging to predict which phase will form. Most current computational studies focus on thermodynamic stability and do not explicitly mention kinetic behavior, leaving a gap in our predictive capabilities for materials discovery. In addition, materials databases are inherently biased toward successful syntheses, often omitting failed attempts, further skewing discovery efforts. A detailed treatment of kinetic obstacles is beyond the scope of this study. Nevertheless, identifying low-energy, dynamically stable structures remains crucial in materials discovery. These candidates define the landscape of potentially accessible compounds and provide valuable guidance for experimental efforts. Even without full kinetic models, exploring these stable structures allows us to uncover new materials and expand the boundaries of known chemical space.

## Conflicts of interest

There are no conflicts to declare.

## Supplementary Material

RA-016-D5RA09680D-s001

## Data Availability

Processed datasets, along with VASP INCAR, POSCAR, and XDATCAR files are publicly available in Zenodo at https://zenodo.org/records/17836343. The archive includes the following: two INCAR files used for the NVT and NVE simulations, structure files (POSCAR) for the three systems, the corresponding MD trajectories file (XDATCAR), and their displacement relative to the equilibrium positions at each time step (Displacement_###K). Supplementary information (SI): additional analyses of the vibrational and dynamical properties of Ce–Co–Cu compounds. Fig. S1 and S2 compares harmonic and AIMD-derived vibrational density of states (DOS) for Ce_10_Co_11_Cu_4_ and Ce_12_Co_7_Cu. Fig. S3 examines the effect of AIMD time-step size on atomic displacement distributions and DOS, while Fig. S4 evaluates the influence of spin polarization on the dynamical properties. See DOI: https://doi.org/10.1039/d5ra09680d.
