# Correction: Anharmonic effects on the dynamical stability of Ce–Co–Cu intermetallic ternary compounds

**DOI:** 10.1039/d6ra90034h

**Published:** 2026-04-02

**Authors:** Wei-Shen Tee, Weiyi Xia, Rebecca Flint, Cai-Zhuang Wang

**Affiliations:** a Ames National Laboratory, U.S. Department of Energy, Iowa State University Ames Iowa 50011 USA; b Department of Physics and Astronomy, Iowa State University Ames Iowa 50011 USA

## Abstract

Correction for ‘Anharmonic effects on the dynamical stability of Ce–Co–Cu intermetallic ternary compounds’ by Wei-Shen Tee *et al.*, *RSC Adv.*, 2026, **16**, 14395–14405, https://doi.org/10.1039/D5RA09680D.

In the original article, the Acknowledgements section was omitted. The Acknowledgements section for this article is given below.

**Acknowledgements**

Work at Ames National Laboratory was supported by the U.S. Department of Energy (DOE), Office of Science, Basic Energy Sciences, Materials Sciences and Engineering Division, including a grant of computer time at the National Energy Research Scientific Computing Center (NERSC), Berkeley, CA. Ames National Laboratory is operated for the U.S. DOE by Iowa State University under Contract No. DE-AC02-07CH11358.

The Royal Society of Chemistry apologises for these errors and any consequent inconvenience to authors and readers.

