# Supplementary material for: Anharmonic effects on the dynamical stability of Ce–Co–Cu intermetallic ternary compounds
Source: RSC Adv. 2026 Mar 17;16(16):14395–405. doi: 10.1039/d5ra09680d (PMC12993825; doi:10.1039/d5ra09680d)
Supplement: RA-016-D5RA09680D-s001 [file RA-016-D5RA09680D-s001.pdf]

Supplementary Information (SI) for RSC Advances.
This journal is © The Royal Society of Chemistry 2026

Anharmonic effects on the dynamical stability of Ce-Co-Cu intermetallic ternary compounds

Supplementary Materials

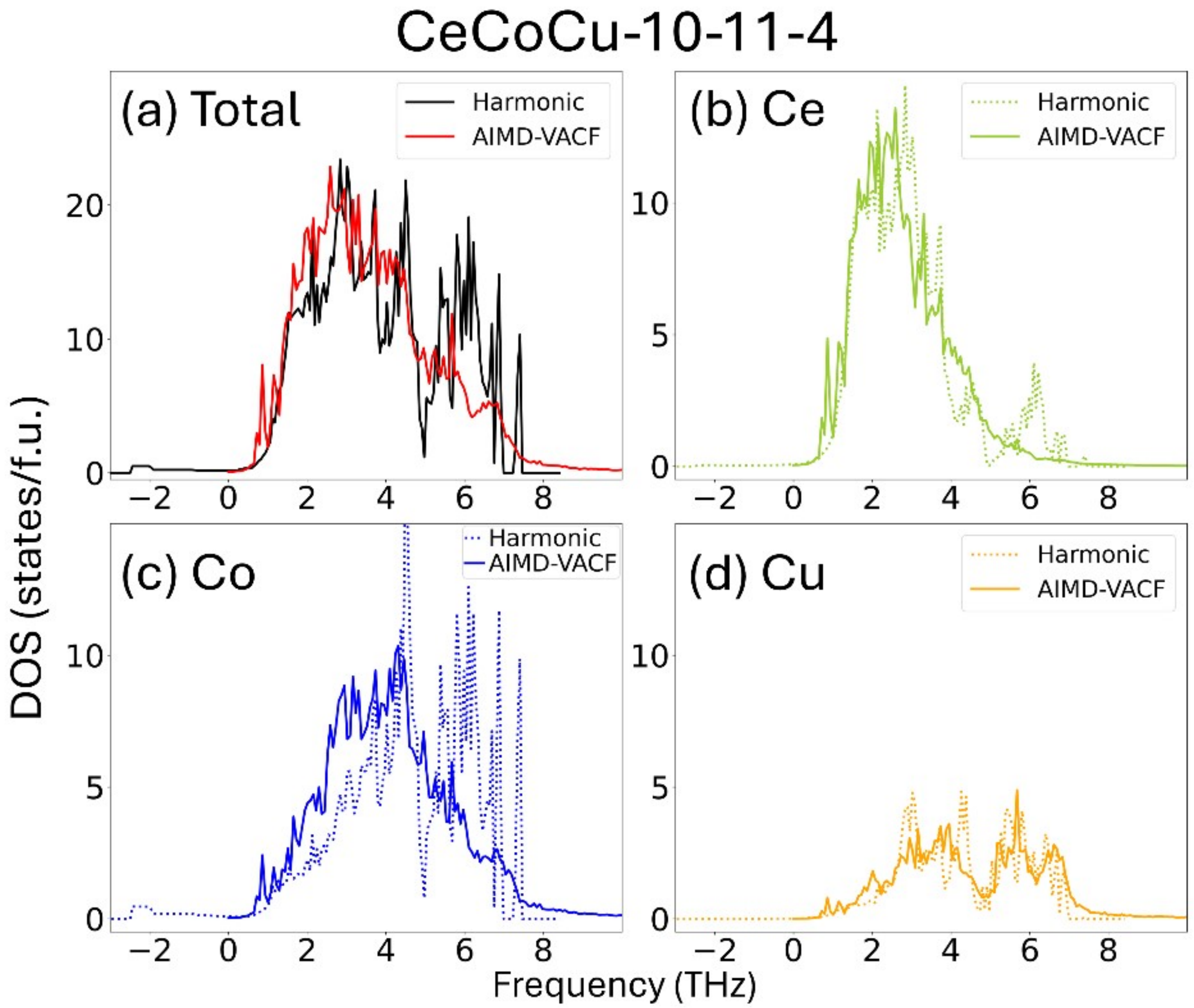

Fig. S1 Total and partial vibrational density of states (DOS) of $Ce_{10}Co_{11}Cu_4$ calculated using the harmonic approximation at 0 K are compared with those from AIMD simulations at 500 K using the velocity autocorrelation function (VACF) method.

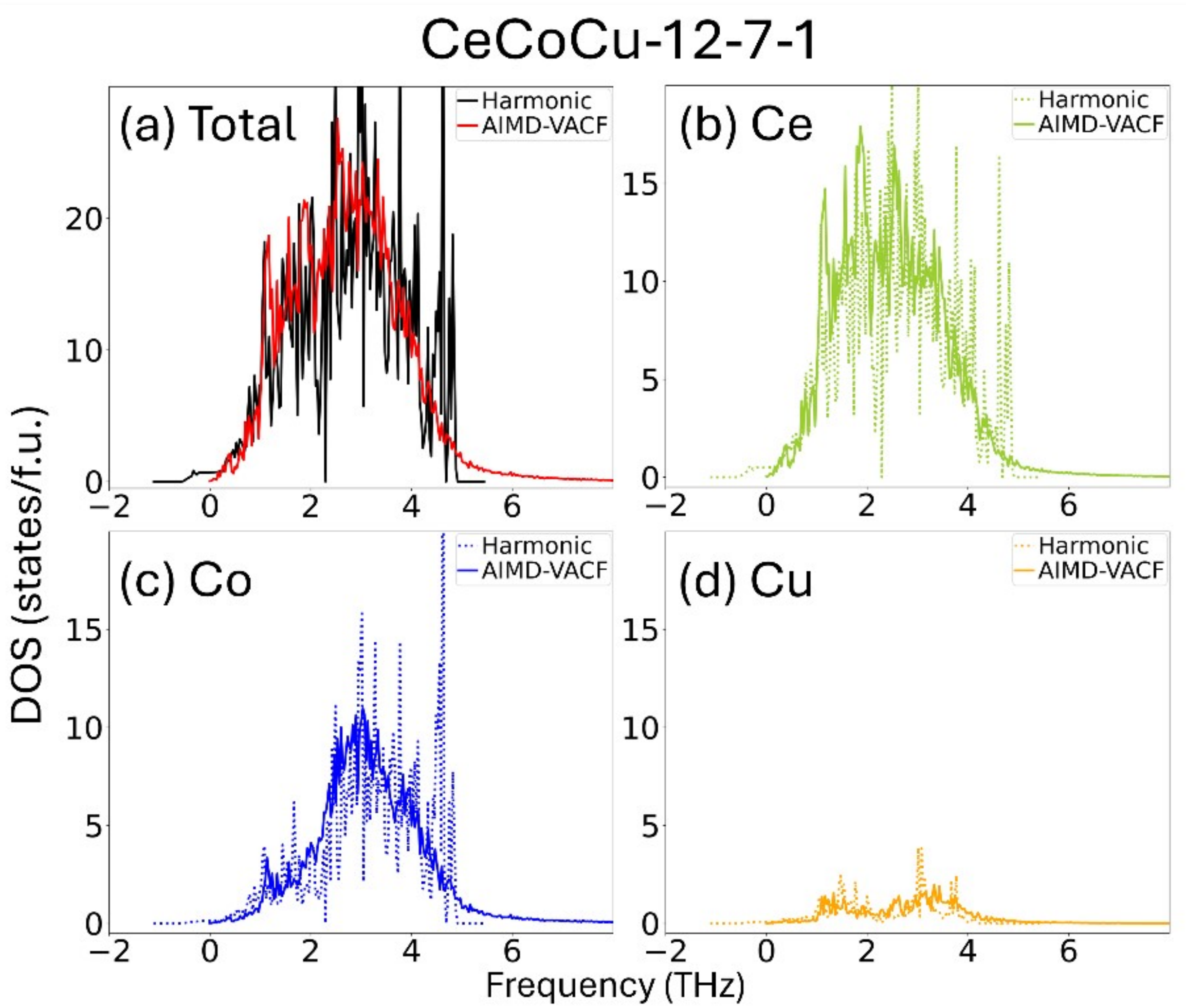

Fig. S2 Total and partial vibrational density of states (DOS) of $Ce_{12}Co_7Cu$ calculated using the harmonic approximation at 0 K are compared with those from AIMD simulations at 500 K using the velocity autocorrelation function (VACF) method.

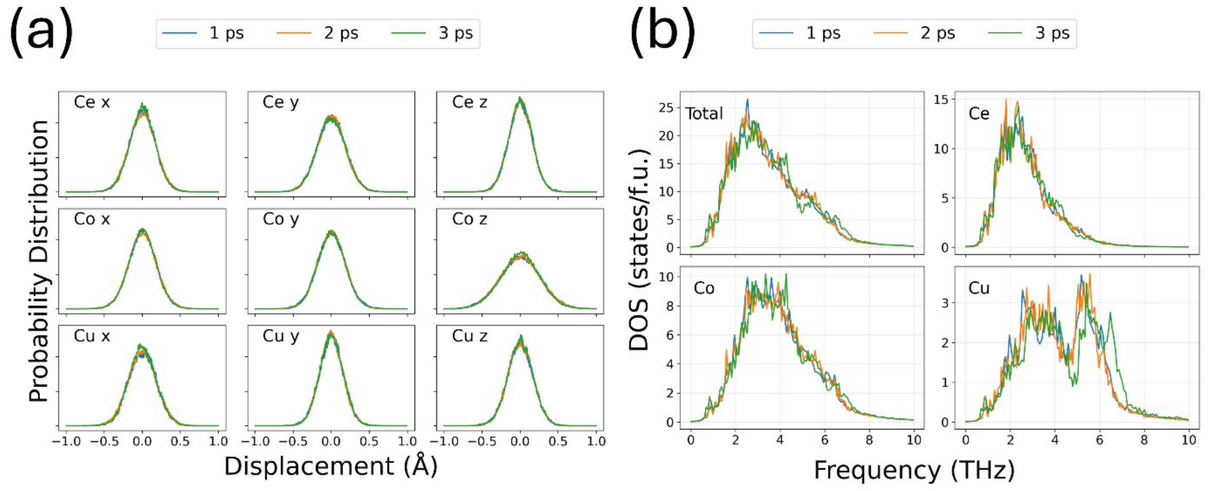

Fig. S3. (a) Probability distribution of atomic displacements and **(b)** vibrational density of states (DOS) for ideal $Ce_{10}Co_{11}Cu_4$ at 800 K (NVT–NVE), calculated using AIMD with time steps of 1 fs, 2 fs, and 3 fs. The comparison demonstrates the effect of time-step size on statistical and vibrational properties.

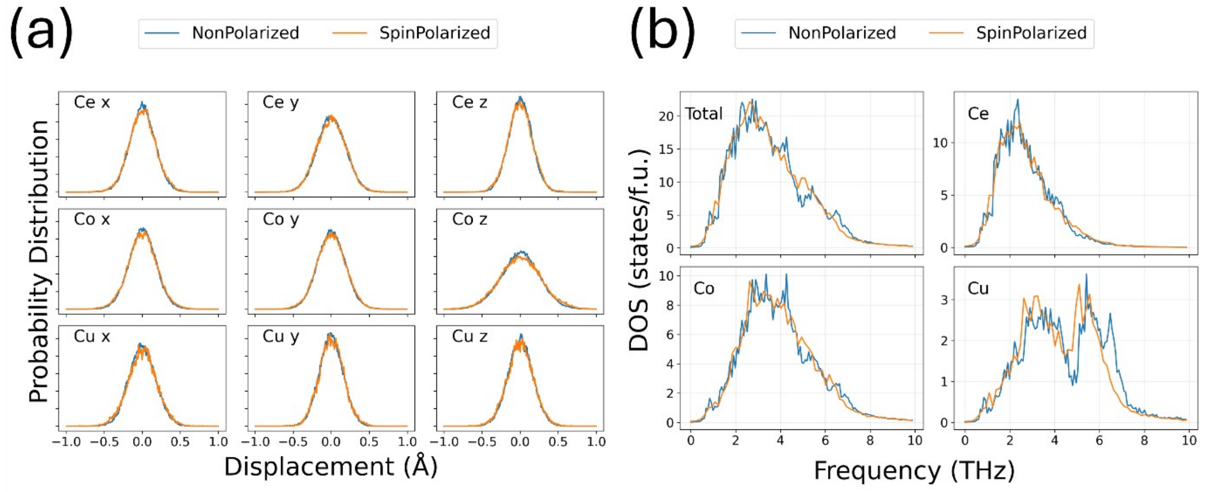

Fig. S4. (a) Probability distribution of atomic displacements and **(b)** vibrational density of states (DOS) for ideal $Ce_{10}Co_{11}Cu_4$ at 800 K (NVT–NVE), computed using spin-polarized and non-spin-polarized AIMD simulations to assess the effect of spin polarization on the dynamical properties.
